# Predicting the Pursuit of Post-Secondary Education: Role of Trait Emotional Intelligence in a Longitudinal Study

**DOI:** 10.3389/fpsyg.2019.01182

**Published:** 2019-05-24

**Authors:** Hiten P. Dave, Kateryna V. Keefer, Samantha W. Snetsinger, Ronald R. Holden, James D. A. Parker

**Affiliations:** ^1^Department of Psychology, University of Western Ontario, London, ON, Canada; ^2^Department of Psychology, Trent University, Peterborough, ON, Canada; ^3^Department of Psychology, Queen's University, Kingston, ON, Canada

**Keywords:** trait emotional intelligence, longitudinal, post-secondary pursuit, emerging adulthood, nationally-representative data

## Abstract

Trait Emotional Intelligence (EI) is a constellation of emotional self-perceptions and dispositions related to perceiving, understanding, using, and managing emotions of self and others. Although higher trait EI has been implicated in post-secondary success among university students. There is lack of evidence for whether it predicts the pursuit of post-secondary education (PSE) in emerging adulthood. This was the first study to investigate the role of trait EI in PSE pursuit using a large, nationally-representative sample of Canadian young adults who participated in the National Longitudinal Survey for Children and Youth (NLSCY). Participants in this dataset reported on their PSE status at three biennial waves (age 20–21, 22–23, and 24–25), and completed a four-factor self-report scale for trait EI (Emotional Quotient Inventory: Mini) at ages 20–21 and 24–25. Higher trait EI subscale scores were significantly associated with greater likelihood of PSE participation both concurrently, and at 2- and 4-year follow-ups. Overall, these associations were larger for men than women. The finding that these links persisted over a multi-year period is particularly promising, as it represents an important validation step toward further investment in socioemotional competencies as part of youth development interventions.

## Introduction

The period of emerging adulthood is an important developmental stage where one's decisions can have substantial personal, social and economic implications for the individual (Arnett, 2000; Lüdtke et al., 2011). One such decision involves the pursuit of post-secondary education (PSE), which is essential for better vocational outcomes in the modern economy (Toutkoushian and Paulsen, 2016; Statistics Canada, 2017; Uppal, 2017). Canadians with a university degree have an employment rate of over 70%, compared to those without one (under 60%, decreasing with lower education levels; Statistics Canada, 2017). PSE completion is also linked to higher income levels in both males and females (Uppal, 2017). However, many young adults who enter PSE leave without completing the program. The attrition rate for high-school graduates transitioning to PSE is ~50% in the United States and Canada (Shaienks et al., 2008; Ross et al., 2012). Moreover, there is a sizeable segment of high school graduates who do not pursue higher education (Finnie, 2012). Understanding the factors that predict PSE pursuit and attainment can inform both policy and programming for enhancing employability of young adults and building a highly skilled workforce of tomorrow.

Models of PSE attainment have traditionally emphasized cognitive metrics—IQ scores, achievement tests, school grades—as key predictors of outcomes. However, cognitive skills alone are no longer considered sufficient for explaining PSE success, with a growing wave of evidence pointing to the unique contributions of non-cognitive factors, such as personality traits, emotional, and interpersonal competencies, and aspects of self-concept (Pascarella and Terenzini, 2005; Richardson et al., 2012; Rowan-Kenyon et al., 2017; Boerchi et al., 2018). Non-cognitive factors are of particular interest from a practical standpoint, because they are more malleable and therefore potentially more amenable to intervention (Richardson et al., 2012; Boerchi et al., 2018).

One of such non-cognitive factors implicated in post-secondary success is trait emotional intelligence, or trait EI (Perera and DiGiacomo, 2013; Parker et al., 2018). Trait EI is defined as a constellation of emotional self-perceptions and dispositions related to perceiving, understanding, using, and managing emotions of self and others (Petrides et al., 2018). Conceptualized as a set of lower-order personality facets, trait EI is assessed via self-report questionnaires and shares moderate degree of overlap with the higher-order Big Five personality traits (Petrides et al., 2007). However, trait EI is not redundant with other constructs, and it has been consistently found to explain incremental variance in various life outcomes beyond measures of cognitive intelligence, mood, and basic personality (Petrides et al., 2007; Wood et al., 2009; Andrei et al., 2016). Trait EI is also distinct from the construct of ability EI (Petrides and Furnham, 2001). Ability EI reflects emotion-related knowledge and the ability to apply that knowledge when instructed to do so; whereas trait EI entails emotional self-efficacy and behavioral dispositions which reflect how a person utilizes their EI abilities in every-day settings (Austin et al., 2008; Mikolajczak, 2009; Keefer, 2015). Given this distinction between aptitude (ability EI) and typical behavior (trait EI), it is not surprising that trait EI has emerged as the stronger and more reliable predictor of various life outcomes than ability EI (Keefer et al., 2018).

A considerable body of research has linked higher trait EI to positive educational outcomes at both secondary and post-secondary levels, including higher grades, fewer school absences, and greater likelihood of degree completion (Humphrey et al., 2007; Perera and DiGiacomo, 2013; Petrides et al., 2018). However, much of this research is cross-sectional, with only a small number of studies utilizing longitudinal designs (e.g., Parker et al., 2004; Keefer et al., 2012). Moreover, extant evidence linking trait EI to long-term PSE outcomes is limited to students who were already enrolled in college or university. To date, there have been no studies examining whether trait EI predicts the likelihood of a young person entering PSE in the first place—an empirical question that requires longitudinal follow-ups of a large representative cohort of youth. The present study capitalized on 4 years of available longitudinal data from the National Longitudinal Survey for Children and Youth (NLSCY; Statistics Canada, 2010a) to investigate the long-term utility of trait EI in predicting PSE pursuit from age 20–21 to 24–25 in a large, nationally-representative sample of young Canadians. To provide more context, the following section reviews existing longitudinal evidence on trait EI in PSE contexts.

## Trait EI in Post-Secondary Contexts

The first series of studies to evaluate the prospective utility of trait EI in predicting post-secondary outcomes of emerging adults transitioning to PSE was the Trent Academic Success and Wellness Project (TASWP; Parker et al., 2018). This research was conducted at a medium-sized Canadian university (Trent University), recruiting four successive cohorts of undergraduate students (total *N* = 3,500) at the beginning of their first year. Participants were administered an array of psychological self-report instruments, including a measure of trait EI called the Emotional Quotient Inventory-Short form (EQ-i:S; Bar-On, 2002), which assesses four trait EI domains: Interpersonal, Intrapersonal, Stress Management, and Adaptability. Participants also consented to releasing their high-school grades and subsequent university records for the project.

In the first TASWP study (Parker et al., 2004) participants' EQ-i:S scores were matched with their academic record at the end of their first year of university. In order to measure academic success, the participants were processed as two groups: academically successful (those with grade-point-averages [GPAs] > 79%) and academically unsuccessful (GPA < 60% for that academic year). Results revealed that, despite having comparable age, course load, and high-school grades, successful students scored significantly higher than the unsuccessful group on total trait EI, as well as on the Intrapersonal, Stress Management, and Adaptability scales assessed by the EQ-i:S. Discriminant Function Analysis classified students into the successful and unsuccessful groups based on their EQ-i:S scores and revealed an overall 86% correct classification rate. This was the earliest compelling evidence showing that trait EI is a relevant factor in PSE settings, as it incrementally predicted academic success independent of a students' previous academic record (Parker et al., 2004).

In a subsequent TASWP study, trait EI was also found to predict student retention into the second year of university (Parker et al., 2006). Again, despite having comparable age, course load, and high-school grades, students who persisted beyond the first year of university had significantly higher trait EI-related competencies (Interpersonal, Intrapersonal, Stress Management, and Adaptability) at the start of the academic year, compared to those who withdrew. This effect was moderated by gender: males who withdrew had significantly lower Interpersonal and total trait EI scores than both males and females who persisted, while females who withdrew had significantly lower total trait EI scores than males who persisted (Parker et al., 2006). Importantly, these initial TASWP findings have been since independently replicated in university samples from Europe and the United States (Parker et al., 2005a; Qualter et al., 2009; Saklofske et al., 2012; Sanchez-Ruiz et al., 2013).

A 6-year follow-up of the TASWP cohorts indicated that first-year trait EI profiles also significantly predicted degree completion status (graduated vs. withdrew): students who left without completing their degree had both significantly lower total trait EI levels and low scores across all four trait EI domains assessed (Keefer et al., 2012). More recently, Parker et al. (2016) reported similar results for a subsample of academically gifted TASWP participants who had a high-school GPA of 90% or better: gifted students who entered university with lower trait EI scores were significantly less likely to graduate within 6 years, compared to their gifted peers with high trait EI. Taken together, this evidence suggests that trait EI is predictive of long-term PSE attainment regardless of students' cognitive intelligence or exceptional academic ability (Parker et al., 2018).

There are several possible mechanisms by which trait EI predicts post-secondary success. One of the more proximal variables is its function as a resilience factor for post-secondary students. Attending a college or university can be a very stressful transition, presenting an array of personal and interpersonal challenges for students (Pascarella and Terenzini, 2005), especially when having to move away from their home town (Witkow et al., 2015). This demographic, comprised mostly of young adults, leave the proximity of pre-existing relationships (like family and friends) and have to form new ones, as well as adapt to a more challenging academic load (Fussell et al., 2007). Compounding this stress is the rising financial costs of attending colleges and universities (Finnie, 2012; Statistics Canada, 2016). This often requires students to take up part-time work, which poses an additional challenge in attempting to balance academic, social, and work life (see Moulin et al., 2013). Consistent with its resilience function, trait EI has been linked with fewer physical fatigue symptoms (Brown and Schutte, 2006; Thompson et al., 2007), lower levels of social anxiety and loneliness (Summerfeldt et al., 2006), and use of more adaptive coping strategies in students (Saklofske et al., 2007). These variables in turn mediate the relationship between trait EI and academic adjustment and performance (Perera and DiGiacomo, 2015).

Three of the main contributing factors in the relationship between trait EI and PSE success are general interpersonal skills, motivation/optimism, and decision-making (Perera and DiGiacomo, 2015). Indeed, all theories of trait EI consider the ability to empathize with others' feelings as one of its core components (Bar-On, 1997; Petrides and Furnham, 2000). This is an important skill for students in an environment requiring collaboration with others (Wang et al., 2009). Students deficient in this competency can feel alienated from campus life—which has been implicated in student attrition (see Wilcox et al., 2005). Higher trait EI is also linked with a more confident and positive outlook on future outcomes (Petrides and Furnham, 2001; Bar-On, 2002). This is an ideal state of mind to persist and attain one's academic goals (Nes and Segerstrom, 2006; Carver and Connor-Smith, 2010), and stay engaged in learning activities (Linnenbrink, 2007). The capacity to effectively collaborate, utilize emotions resourcefully in problem-solving, and stay determined while adaptively coping with socioemotional and academic challenges is a psychological profile typically linked with post-secondary success (Credé and Niehorster, 2012).

## The Present Study

As is evident from the studies reviewed above, extant research on the role of trait EI in PSE attainment has been limited to an overly restricted participant pool—one in which everyone has already made it into a PSE program. Whether trait EI predicts the likelihood of pursuing PSE in the first place is an important question that is yet to be addressed empirically. While it is true that external factors, such as socioeconomic status can influence PSE pursuit, population-based evidence suggests that 43% of Canadians report no barriers in the decision to pursue PSE (irrespective of whether they pursue it or not), compared to 25% citing financial barriers (Finnie, 2012). Therefore, PSE pursuit depends on individual and sociocultural factors more so than on economic barriers alone (Abada and Tenkorang, 2009; Finnie, 2012). Given the importance of PSE in the marketplace, and the demonstrated long-term links between trait EI and PSE success, it is worth investigating if trait EI in emerging adulthood is also associated with the pursuit of higher education. Obtaining empirical evidence for this link is an important validation step toward further investment in trait EI interventions.

The main goal of the current study was to examine the predictive utility of trait EI for PSE pursuit over a 4-year period of emerging adulthood (age 20–21 to 24–25). To accomplish this, we analyzed three biennial waves of nationally-representative data from the Canadian National Longitudinal Survey of Children and Youth (NLSCY; Statistics Canada, 2010a).

The inclusion of multi-year trait EI data is a unique feature of the NLSCY, relative to other comparable databases, such as the Canadian Youth in Transition Survey or the American National Longitudinal Survey of Youth. It is therefore the only current source of longitudinal, population-level data on trait EI. Specifically, the study dataset includes measures of trait EI for 20–21-year-olds and 24–25-year-olds (among its emerging adult demographic). Education data for that sample was available concurrently, and prospectively at age 22–23 and 24–25. We expected trait EI to positively predict PSE participation in emerging adulthood. In line with previous research on trait EI in PSE contexts (e.g., Parker et al., 2004, 2011), gender was included as a potential moderator in these analyses.

## Methods

### Data Source

This study utilized a stratified, nationally representative, multi-wave dataset from Statistics Canada called the National Longitudinal Survey of Children and Youth (NLSCY). The survey was administered to children aged 0–11 years old at the start (in 1994), with follow-ups conducted every 2 years from then until 2008, producing a total of 8 cycles. The survey assessed a variety of constructs from demographic information to physical and mental health, educational and vocational outcomes, and socioemotional competencies from early childhood into young adulthood (Statistics Canada, 2010a).

The original Cycle 1 cohort included almost 23,000 children selected to represent the population of all Canadian children aged 0–11 years. Exclusionary criteria included children living in Northwestern territories, or having parents that are either institutionalized, living in First Nations Reserves and Crown Lands, and/or children whose parents were full-time members of the Canadian Armed Forces (representing 2% of the population). Budget restrictions and the need to reduce response burden on households with more than 2 children reduced the sample size to 16,000 in Cycle 2. Close to 10,000 of that sample participated in the final cycle of the survey (Cycle 8).

As this research is based on anonymized secondary data files provided by Statistics Canada, an ethics approval was not required for this particular investigation. We refer readers to the Statistics Act (http://laws-lois.justice.gc.ca/eng/acts/S-19/PITIndex.html) for ethical guidelines that the Government of Canada abided by during data collection.

### Study Sample

For this study we only used data from Cycle 6 (2004), Cycle 7 (2006), and Cycle 8 (2008) of the multi-wave study, as these were the only cycles containing our target age group (early adulthood) along with the relevant variables (trait EI and PSE pursuit). We specifically used a subsample of ~1,400 Canadian youth (50% males) aged 20–21 who had completed the same measure of trait EI (EQ-i: Mini) at Cycle 6, and again at Cycle 8 when they were 24–25 years old. The EQ-i: Mini data was not available at any other Cycle for this cohort. PSE information for this cohort was available at Cycles 6, 7, and 8. Participants were surveyed via face-to-face or telephone interview conducted by a trained Statistics Canada interviewer using computer-assisted interviewing (Statistics Canada, 2010b). The NLSCY data User Guide contains more information on the sampling design and data collection procedure (Statistics Canada, 2010a).

### Measures

#### Trait EI

Trait EI was assessed using a shortened, 20-item version of the EQ-i: Short (Bar-On, 2002) called the EQ-i: Mini. For the present sample, this measure was only administered to participants at Cycle 6 (age 20–21) and Cycle 8 (age 24–25). The EQ-i: Mini consists of self-referential statements rated by participants on a 5-point Likert scale (from 1 = “Very seldom true or not true” to 5 = “Very often true or true”). Higher scores imply higher levels of trait EI. Of the 20 items, 16 tap into dimensions of interest (Interpersonal, Intrapersonal, Stress Management and Adaptability) while the other 4 are screeners for participants' General Mood. As General Mood is not part of the trait EI construct, it was not considered in the present study. For a full list of items see Appendix A in Supplementary Material.

This measure demonstrated good reliability coefficients: for each dimension of the EQ-i: Mini, average item reliabilities (*R*^2^) and mean inter-item correlations (MICs) across both cycles were moderate: Interpersonal (*R*^2^ = 0.34; MIC = 0.34), Intrapersonal (*R*^2^ = 0.28; MIC = 0.33), Stress Management (*R*^2^ = 0.35, MIC = 0.33), and Adaptability (*R*^2^ = 0.35; MIC = 0.31). The EQ-i: Mini also had good Cronbach's alpha coefficients for the total scores at each time-point (0.76 at Cycle 6 and 0.77 at Cycle 8).

#### PSE Participation

A variable in the NLSCY dataset coded participants' self-reported educational status at each Cycle by the following categories: 01 = School leaver; 02 = In high school; 03 = Completed high school but not in post-secondary; 04 = In post-secondary; 05 = Completed post-secondary; 06–99 = Current education status unknown.

For the present study, this variable was re-coded to form a dichotomous variable with the following groups: No PSE (categories 01, 02, and 03) and Some PSE (categories 04 and 05), respective to that specific time-point. Cases with unknown education status (06−99) were excluded from the analyses.

To examine change in PSE status across time-points, the education variables were re-coded as a three-level variable: No PSE at all (at either time-point), New PSE (for those who had no PSE at a previous time-point but acquired some PSE by a second time-point), and Previous PSE (for those who had some PSE at a previous time-point).

### Statistical Analyses

#### Trait EI and PSE Pursuit

A series of between-groups (for concurrent) and within-subjects (for prospective) Multivariate Analyses of Variance (MANOVAs) were conducted with the PSE groups (No PSE and Some PSE for PSE status; No PSE, New PSE, and Previous PSE for change in PSE status) as the grouping independent variable and the EQ-i: Mini subscales (Interpersonal, Intrapersonal, Stress Management, and Adaptability) as dependent variables. Gender was also included as a second grouping variable in the analyses to assess for potential moderating effects. These MANOVAs were conducted using the latest version of SPSS software.

#### Significance Criteria

Following the NLSCY guidelines (Statistics Canada, 2010a), all analyses were conducted on weighted data, calculated from longitudinal sampling weights (pre-generated by Statistics Canada), which are adjusted for longitudinal survey-wide non-response and post-stratified to known frequencies by age, sex, and province to reflect the original survey population (Statistics Canada, 2010a). This allows for making population-based inferences from the results. However, the weighting procedure inflates the effective sample sizes (to the hundreds of thousands) to a point where orthodox statistical significance testing (*p* < 0.05) is not applicable^1^. Therefore, we interpreted the results based on standardized effect size measures, such as the Eta statistic (η; the square root of Partial Eta-Squared values in SPSS) for mean differences, and Cramer's V for cross-tabulations of frequencies. These values provide information on the magnitude of the effect, and are independent of the sample size (Ferguson, 2009; Gignac and Szodorai, 2016). There is no “objective” indicator for the extent of difference required to be deemed “non-trivial.” Therefore, we followed the guideline that effect size benchmarks for “practically significant” effects should be determined based on evidence from the specific research context (Hill et al., 2008; Keefer et al., 2013). Our interpretations relied on empirically derived benchmarks used in previous studies on socioemotional competencies (Durlak et al., 2011; Keefer et al., 2013). Specifically, effect sizes of ≥0.10 (non-squared) units were considered to be non-trivial, or practically significant.

## Results

### Concurrent Associations With PSE Status at Age 20–21

Of the total study sample who had valid EQ-i: Mini data at Cycle 6, 66% reported at least some PSE experience at Cycle 6, 22% reported no PSE experience at Cycle 6, and the remaining 12% had unknown or missing Cycle 6 education status. Respondents who had unknown/missing vs. valid Cycle 6 education data were compared on their Cycle 6 EQ-i: Mini subscale scores and proportions of men and women. Missingness of Cycle 6 education data was not significantly associated with gender (Cramer's V = 0.06) or Cycle 6 EQ-i: Mini scores (Wilk's lambda = 0.99, Eta = 0.08). Only the respondents with valid Cycle 6 education data were used in subsequent analyses of variance.

A 2 × 2 factorial MANOVA was conducted on Cycle 6 (age 20–21) EQ-i: Mini subscale scores, using Cycle 6 PSE Status (some PSE vs. no PSE) and gender (men vs. women) as the grouping factors. There was a significant multivariate effect of PSE Status (Wilk's lambda = 0.97, Eta = 0.18), but the moderating effect of gender was non-significant (Wilk's lambda = 0.99, Eta = 0.08). Eta values and contrasts of group means from follow-up univariate ANOVAs are presented in Table 1. Individuals who had at least some PSE experience by age 20–21 had significantly higher concurrent scores on all four EQ-i: Mini subscales than individuals who had no PSE experience.

**Table 1:** Concurrent and Prospective Associations between EQ-i: Mini Subscale Scores and PSE Status.

**Effects**	**Interpersonal at age 20–21**	**Intrapersonal at age 20–21**	**Stress Mngt. at age 20–21**	**Adaptability at age 20–21**
**PSE STATUS AT AGE 20–21**				
*Eta* PSE	0.11^*^	0.12^*^	0.15^*^	0.14^*^
*Eta* PSE × Gender	0.07	0.03	0.00	0.07
*M* (*SD*) Some PSE	4.30 (0.49)^a^	3.77 (0.72)^a^	3.94 (0.69) ^a^	4.03 (0.57)^a^
*M* (*SD*) No PSE	4.08 (0.72)^b^	3.55 (0.84)^b^	3.67 (0.82)^b^	3.82 (0.70)^b^
**PSE STATUS AT AGE 22–23**				
*Eta* PSE	0.14^*^	0.05	0.15^*^	0.05
*Eta* PSE × Gender	0.15^*^	0.09^†^	0.06	0.13^*^
*M* (*SD*) Some PSE Men	4.13 (0.61)^a^	3.74 (0.74)^a^	3.96 (0.67)^a^	3.99 (0.56)^a^
*M* (*SD*) No PSE Men	3.69 (0.53)^b^	3.44 (0.78)^b^	3.53 (0.87)^b^	3.69 (0.61)^b^
*M* (*SD*) Some PSE Women	4.46 (0.52)^c^	3.76 (0.72)^a^	3.94 (0.68)^a^	4.01 (0.58)^a^
*M* (*SD*) No PSE Women	4.44 (0.41) ^c^	3.85 (0.91) ^a^	3.76 (0.81) ^c^	4.13 (0.74)^a^
**PSE STATUS AT AGE 24–25**				
*Eta* PSE	0.22^*^	0.09^†^	0.11^*^	0.13^*^
*Eta* PSE × Gender	0.16^*^	0.10^*^	0.11^*^	0.00
*M* (*SD*) Some PSE Men	4.15 (0.54)^a^	3.76 (0.73)^a^	4.00 (0.69)^a^	4.04 (0.54)^a^
*M* (*SD*) No PSE Men	3.60 (0.52)^b^	3.33 (0.80)^b^	3.54 (0.79)^b^	3.71 (0.60)^b^
*M* (*SD*) Some PSE Women	4.45 (0.42)^c^	3.76 (0.74)^a^	3.91 (0.70)^a^	4.00 (0.60)^a^
*M* (*SD*) No PSE Women	4.35 (0.54)^c^	3.78 (0.89)^a^	3.91 (0.73)^a^	3.88 (0.77)^b^
	**Interpersonal at age 4–25**	**Intrapersonal at age 24–25**	**Stress Mngt. at age 24–25**	**Adaptability at age 24–25**
**PSE STATUS AT AGE 24–25**				
*Eta* PSE	0.17^*^	0.09^†^	0.11^*^	0.15^*^
*Eta* PSE × Gender	0.06	0.09^†^	0.04	0.12^*^
*M* (*SD*) Some PSE Men	4.22 (0.57)^a^	3.79 (0.67)^a^	3.95 (0.71)^a^	4.19 (0.54)^a^
*M* (*SD*) No PSE Men	3.86 (0.52)^b^	3.42 (0.67)^b^	3.63 (0.75)^b^	3.72 (0.59)^b^
*M* (*SD*) Some PSE Women	4.52 (0.44)^c^	3.85 (0.69)^a^	3.97 (0.69)^a^	4.19 (0.61)^a^
*M* (*SD*) No PSE Women	4.35 (0.63)^a^	3.84 (0.74)^a^	3.83 (0.77)^c^	4.14 (0.76)^a^

Mean scores on the EQ-i: Mini subscales range from 1 to 5. Subscale means with different superscripts are significantly different from each other. PSE, Post-secondary education;

* Non-trivial effect;

† *Marginal effect*.

### Prospective 2-Year Associations With PSE Status at Age 22–23

Of the total study sample who had valid EQ-i: Mini data at Cycle 6, 22% did not return for Cycle 7 (age 22–23). Of the returning Cycle 7 sample, 73% reported at least some PSE experience at Cycle 7, 14% reported no PSE experience at Cycle 7, and the remaining 13% had unknown or missing Cycle 7 education status. Respondents who had unknown/missing vs. valid Cycle 7 education data were compared on their Cycle 6 EQ-i: Mini subscale scores, proportions of men and women, and Cycle 6 PSE Status (some vs. none). Missingness of Cycle 7 education data was not significantly associated with gender (Cramer's V = 0.09) or Cycle 6 EQ-i: Mini scores (Wilk's lambda = 1.0, Eta = 0.06). However, missingness was significantly associated with Cycle 6 PSE Status (Cramer's V = 0.18); respondents who reported no PSE at Cycle 6 were three times more likely (18%) to have missing Cycle 7 PSE data than respondents who reported some PSE at Cycle 6 (6%). Only the respondents with valid Cycle 7 education data were used in subsequent analyses of variance.

A 2 × 2 MANOVA was conducted on Cycle 6 (age 20–21) EQ-i: Mini subscale scores, using Cycle 7 (age 22–23) PSE Status (Some PSE vs. No PSE) and gender (men vs. women) as the grouping factors. There was a significant multivariate effect of PSE Status (Wilk's lambda = 0.96, Eta = 0.20), as well as a significant moderation effect of gender (Wilk's lambda = 0.96, Eta = 0.19). Eta values and contrasts of group means from follow-up univariate ANOVAs are presented in Table 1. Individuals (both men and women) who had at least some PSE experience at age 22–23 had significantly higher Stress Management scores at age 20–21 than individuals who had no PSE experience at age 22–23. In addition, men who had at least some PSE experience at age 22–23 had significantly higher Interpersonal, Intrapersonal, and Adaptability scores at age 20–21 than men who had no PSE experience at age 22–23; for women, these effects were non-significant.

### Predicting Change in PSE Status From Age 20–21 to 22–23

Of the respondents who had reported Cycle 7 education data, 76% reported at least some PSE experience previously in Cycle 6, 8% reported no PSE experience in either Cycle 6 or Cycle 7, and 8% reported no PSE experience at Cycle 6 but had newly acquired some PSE experience by Cycle 7.

A 3 × 2 factorial MANOVA was conducted on Cycle 6 (age 20–21) EQ-i: Mini subscale scores, using Cycle 7 (age 22–23) PSE Status Change (Previous PSE vs. New PSE vs. No PSE) and gender (men vs. women) as the grouping factors. There was a significant multivariate effect of PSE Status Change (Wilk's lambda = 0.94, Eta = 0.17), as well as a significant moderation effect of gender (Wilk's lambda = 0.96, Eta = 0.14). Eta values and contrasts of group means from follow-up univariate ANOVAs are presented in Table 2. Among those men who had no PSE experience at age 20–21, higher Interpersonal and Intrapersonal scores at age 20–21 differentiated the individuals who acquired new PSE experience by age 22–23 from individuals who did not. For women, none of the EQ-i: Mini subscales at age 20–21 predicted new acquisition of PSE experience by age 22–23.

**Table 2:** Prospective Associations between EQ-i: Mini Subscale Scores and PSE Status Change.

**Effects**	**Interpersonal at age 20–21**	**Intrapersonal at age 20–21**	**Stress Mngt. at age 20–21**	**Adaptability at age 20–21**
**PSE STATUS CHANGE FROM AGE 20–21 TO 22–23**				
*Eta* PSE Change	0.16^*^	0.06	0.18^*^	0.09^†^
*Eta* PSE Change × Gender	0.15^*^	0.09^†^	0.08	0.12^*^
**MEN**				
*M* (*SD*) Previous PSE	4.16 (0.51)^a^	3.74 (0.74)^a^	3.99 (0.65)^a^	4.03 (0.54)^a^
*M* (*SD*) New PSE	3.94 (1.13)^b^	3.89 (0.68)^a^	3.59 (0.76)^b^	3.82 (0.69)^b^
*M* (*SD*) No PSE	3.69 (0.53) ^c^	3.44 (0.78) ^b^	3.53 (0.88) ^b^	3.70 (0.62) ^b^
**WOMEN**				
*M* (*SD*) Previous PSE	4.44 (0.40)^d^	3.76 (0.72)^a^	3.96 (0.66)^a^	4.02 (0.57)^a^
*M* (*SD*) New PSE	4.36 (0.56)^d^	3.74 (0.79)^a^	3.77 (0.91)^c^	3.91 (0.69)^a^
*M* (*SD*) No PSE	4.44 (0.55)^d^	3.83 (0.98)^a^	3.78 (0.77)^c^	4.11 (0.77)^a^
**PSE STATUS CHANGE FROM AGE 22–23 TO 24–25**				
*Eta* PSE Change	0.21^*^	0.08	0.09^†^	0.12^*^
*Eta* PSE Change × Gender	0.20^*^	0.15^*^	0.10^*^	0.15^*^
**MEN**				
*M* (*SD*) Previous PSE	4.18 (0.53)^a^	3.73 (0.72)^a^	4.03 (0.65)^a^	4.04 (0.52)^a^
*M* (*SD*) New PSE	3.92 (0.58)^b^	3.20 (1.30)^b^	4.11 (0.78)^a^	3.39 (0.70)^b^
*M* (*SD*) No PSE	3.52 (0.54)^c^	3.27 (0.78)^b^	3.61 (0.82)^b^	3.66 (0.65)^c^
**WOMEN**				
*M* (*SD*) Previous PSE	4.45 (0.42)^d^	3.75 (0.74)^a^	3.94 (0.68)^a^	3.99 (0.70)^a^
*M* (*SD*) New PSE	4.55 (0.46)^d^	4.28 (0.71)^c^	3.63 (0.66)^b^	4.23 (0.63)^d^
*M* (*SD*) No PSE	4.42 (0.57)^d^	3.83 (0.99)^a^	3.94 (0.74)^a^	3.96 (0.79)^a^

Mean scores on the EQ-i: Mini subscales range from 1 to 5. Subscale means with different superscripts are significantly different from each other. PSE, post-secondary education;

* Non-trivial effect;

† *Marginal effect*.

### Prospective 4-Year Associations With PSE Status at Age 24–25

Of the total study sample who had valid EQ-i: Mini data at Cycle 6, 29% did not return for Cycle 8. Of the returning Cycle 8 sample, 77% reported at least some PSE experience at Cycle 8, 12% reported no PSE experience at Cycle 8, and the remaining 11% had unknown or missing Cycle 8 education status. Respondents who had unknown/missing vs. valid Cycle 8 education data were compared on their Cycle 6 EQ-i: Mini subscale scores, proportions of men and women, and Cycle 7 PSE Status (some vs. none). Missingness was not significantly associated with gender (Cramer's V = 0.09) or Cycle 6 EQ-i: Mini scores (Wilk's lambda = 1.0, Eta = 0.05). However, missingness was significantly associated with Cycle 7 PSE Status (Cramer's V = 0.11); respondents who reported no PSE at Cycle 7 were three times more likely (11%) to have missing Cycle 8 PSE data than respondents who reported some PSE at Cycle 7 (4%). Only the respondents with valid Cycle 8 education data were used in subsequent analyses of variance.

A 2 × 2 factorial MANOVA was conducted on Cycle 6 (age 20–21) EQ-i: Mini subscale scores, using Cycle 8 (age 24–25) PSE Status (some vs. none) and gender (men vs. women) as the grouping factors. There was a significant multivariate effect of PSE Status (Wilk's lambda = 0.95, Eta = 0.24), as well as a significant moderation effect of gender (Wilk's lambda = 0.96, Eta = 0.20). Eta values and contrasts of group means from follow-up univariate ANOVAs are presented in Table 1. Individuals (both men and women) who had at least some PSE experience at age 24–25 had significantly higher Adaptability scores at age 20–21 than individuals who had no PSE experience at age 24–25. In addition, men who had at least some PSE experience at age 24–25 had significantly higher Interpersonal, Intrapersonal, and Stress Management scores at age 20–21 than men who had no PSE experience at age 24–25; these effects were non-significant for women.

### Predicting Change in PSE Status From Age 22–23 to 24–25

Of the respondents who had reported Cycle 8 education data, 86% reported at least some PSE experience previously in Cycle 7, 11% reported no PSE experience in either Cycle 7 or Cycle 8, and 3% reported no PSE experience at Cycle 7 but had newly acquired some PSE experience by Cycle 8. Given the very small percentage of individuals in the last category (corresponding unweighted *n* < 30), the following results should be treated as preliminary.

A 3 × 2 factorial MANOVA was conducted on Cycle 6 EQ-i: Mini subscale scores, using Cycle 8 PSE Status Change (Previous vs. New vs. None) and gender (men vs. women) as the grouping factors. This omnibus test revealed a significant multivariate effect of PSE Status Change (Wilk's lambda = 0.95, Eta = 0.15), as well as a significant moderation effect of gender (Wilk's lambda = 0.92, Eta = 0.20). Eta values and contrasts of group means from follow-up univariate ANOVAs are presented in Table 2. The results differed for men and women. Among those men who had no PSE experience at age 22–23, higher Intrapersonal and Stress Management scores, but lower Adaptability scores at age 20–21 differentiated the men who acquired new PSE experience by age 24–25 from the men who did not. Among those women who had no PSE experience at age 22–23, higher Intrapersonal and Adaptability scores, but lower Stress Management scores at age 20–21 differentiated the women who acquired new PSE experience by age 24–25 from the women who did not.

### Concurrent Associations With PSE Status at Age 24–25

A 2 × 2 MANOVA was conducted on Cycle 8 (age 24–25) EQ-i: Mini subscale scores, using Cycle 8 (age 24–25) PSE Status (Some PSE vs. No PSE) and gender (men vs. women) as the grouping factors. This omnibus test revealed a significant multivariate effect of PSE Status (Wilk's lambda = 0.95, Eta = 0.22), as well as a significant moderation effect of gender (Wilk's lambda = 0.98, Eta = 0.13). Eta values and contrasts of group means from follow-up univariate ANOVAs are presented in Table 1. Individuals (both men and women) who had at least some PSE experience at age 24–25 had significantly higher concurrent Interpersonal and Stress Management scores than individuals who had no PSE experience at age 24–25. In addition, men who had at least some PSE experience at age 24–25 had significantly higher concurrent Adaptability and Intrapersonal scores than men who had no PSE experience at age 24–25; these effects were non-significant for women.

## Discussion

This study was the first to examine the concurrent and longitudinal associations of trait EI with PSE participation using a unique, nationally representative sample of Canadian young adults. Higher trait EI scores (as measured by the EQ-i: Mini) at age 20–21 predicted greater PSE participation both concurrently, as well as prospectively at age 22–23 and 24–25. All four trait EI dimensions were positively associated with PSE participation for both genders concurrently at age 20–21. Longitudinally, all four trait EI dimensions (at age 20–21) were positively associated with PSE participation in men at each time point. Meanwhile, women's PSE pursuit at age 22–23 and 24–25 was only associated with their age 20–21 Stress Management and Adaptability scores. Concurrent PSE status at age 24–25 was positively associated with all four trait EI dimensions for men and with the Interpersonal and Stress Management scores for women. Most importantly, trait EI at age 20–21 significantly predicted acquisition of new PSE over-time. Among men with no PSE at age 20–21, those with higher Interpersonal scores were more likely to enter PSE either 2 or 4 years later. Acquiring new PSE was also predicted by higher Intrapersonal and Stress Management scores for men, and higher Intrapersonal and Adaptability scores for women.

With these findings it is possible to conclude that higher trait EI has non-trivial prospective utility for predicting PSE pursuit during the period of emerging adulthood. These findings also add to the existing body of research linking higher trait EI with success in PSE settings. Students who enter university with higher trait EI levels are more likely to persist beyond first year (Parker et al., 2006), attain higher grades (Parker et al., 2004, 2005a), suffer from less anxiety and use better coping strategies (Summerfeldt et al., 2006; Austin et al., 2010; Saklofske et al., 2012), and are more likely to graduate (Keefer et al., 2012; Parker et al., 2016). Trait EI therefore serves not only as a resilience factor for students already in PSE, but also as an antecedent to pursuing this important life goal in the first place.

There are several possible mechanisms through which trait EI might contribute to greater rates of PSE participation. Individuals with higher trait EI levels may be more likely to pursue PSE because they are more likely to meet admission eligibility criteria due to their better high school performance (Petrides et al., 2004; Perera and DiGiacomo, 2013). In addition, trait EI has been studied in relation to career decision-making process, signifying the importance of these socioemotional competencies in being able to handle the overwhelming nuances of important life decisions (Brown et al., 2003; Avsec, 2012; Di Fabio and Kenny, 2012). A similar mechanism could be influencing the decision to pursue higher education. In today's unpredictable economic times, career decisions require a firmer grasp of nuance, as well as a reasonable level of emotional awareness and stability (Krieshok et al., 2009). Higher levels of trait EI entail factors, such as considering all possibilities before making a decision, and being aware of one's own emotional states (Bar-On, 1997, 2002). These traits have demonstrated to be effective mediators for combatting indecisiveness, lack of information on future opportunities, and productive use of information to make a career decision (Di Fabio and Palazzeschi, 2008, 2009; Di Fabio et al., 2012; Di Fabio and Saklofske, 2014). Trait EI is also positively associated with more adaptive decision-making styles (Di Fabio and Palazzeschi, 2008; Di Fabio and Blustein, 2010). Indeed, attaining a college or university degree is undoubtedly important for better vocational prospects (Toutkoushian and Paulsen, 2016; Statistics Canada, 2017; Uppal, 2017) and would therefore be a crucial factor in career decisions. It is therefore very plausible to hypothesize that if trait EI is implicated in the general process of career decision-making, it may also be associated with the decision to pursue PSE via a similar mechanism. Future research on trait EI and PSE pursuit should consider including decision-making variables as mediating factors.

### Magnitude of Effects

It is important to note that although most of the reported effect sizes were relatively small, close to the 0.10 benchmark (Gignac and Szodorai, 2016), this is still non-trivial when viewed in light of population-based research and the practical significance of the outcome variable. For example, Mikolajczak et al. (2015) studied the effects of trait EI on physical health variables (e.g., doctor visits, days spent in hospital, reimbursed drugs) in a nationally-representative European sample. Similar to the present study, they also found small but meaningful effects (<0.20) when assessing the relationship between high trait EI levels and better physical health outcomes. In arguing for the practical significance of their findings, they bring up the fiscal reality that the “population with below-average socioemotional competencies cost nearly 2 billion more to the Belgian social security, than the population with above-average [trait EI]” (Mikolajczak et al., 2015; p. 12). From the standpoint of public policy, this is considered a worthwhile return on investment (Mikolajczak and Van Bellegem, 2017).

Another economic impact study showed that the implementation of socioemotional programs in schools estimates an economic return of $11 per dollar invested in these programs (Belfield et al., 2015). This is further supported by Durlak et al.'s (2011) meta-analysis indicating that while the implementation of socioemotional learning programs had relatively small mean effect on academic performance (*r* = 0.13), it translated to an overall 11 percentile point gain in grades. Therefore, the present study's relatively small magnitudes of effects for trait EI dimensions on PSE pursuit are hence not at all trivial and, when viewed from a population-based lens, can carry significant economic implications.

In discussing the issue of effect sizes, Keefer et al. (2018) further argued that a broad life outcome, such as PSE attainment is necessarily the product of numerous individual and contextual factors; thus, any single factor can only be expected to account for a small portion of criterion variance by itself. This argument is particularly relevant in the present study, where the effects of trait EI on PSE pursuit were further moderated by gender and trait EI domain.

### Gender and Domain Differences

Some trait EI domains are more gendered than others. Specifically, women tend to score higher than men on the Interpersonal dimension, whereas men tend to score higher than women on Adaptability (Keefer, 2015). Gender differences in the Intrapersonal and Stress Management domains tend to be smaller or inconsistent. Siegling et al. (2012) investigated the nuances of gender differences in trait EI domains in relation to the gender-linked personality traits of communion and agency. Communion refers to compassion, nurturance, or placing a strong salience on interpersonal relationships. Agency involves assertiveness, competitiveness, and self-autonomy. Typically, agentic traits are associated with males while communal traits are associated with females (Bakan, 1966). Siegling et al. (2012) found that gender differences in the Interpersonal vs. Adaptability domains of trait EI were linked to communion vs. agency orientations, respectively.

In our results, the relatively gender-neutral EI traits (i.e., Intrapersonal and Stress Management) were linked to long-term PSE pursuit in both men and women. However, the two gender-typed domains (i.e., Interpersonal and Adaptability) had unique patterns of predictive utility for the counter-stereotypical gender. Controlling for previous PSE status, the Adaptability scores (male-typed traits) predicted long-term gains in PSE status for women, while the Interpersonal scores (female-typed traits) predicted long-term gains in PSE status for men. This differential pattern of relationships provides support for the idea that an androgynous trait EI profile, characterized by high levels of both male-typed and female-typed traits, may be maximally advantageous for both men and women (Keefer, 2015).

Overall, the associations of trait EI with PSE pursuit were stronger and more robust for men than for women. The weaker effects for women may be partially explained by the restricted range of the PSE variable: in the present study the proportion of women pursuing PSE was significantly higher than men (e.g., at age 20–21 the group with “Some PSE” had 81% women). However, similar gender moderation effects have been reported for other criterion variables, showing that trait EI matters more for men than women. For example, Karakuş (2013) investigated trait EI and negative feelings in a sample of primary school teachers. It was found that trait EI directly predicted stress and indirectly (mediated via stress) predicted anxiety levels in male teachers, but this effect was not significant for women. This is consistent with gender differences in the effects of trait EI on stress found by Petrides and Furnham (2006) in a similar workplace study. It is reasoned that men and women are exposed to different forms of stress; specifically, women face other responsibilities, such as family responsibilities and the “glass ceiling effect” of progressing in the organizational hierarchy (Cotter et al., 2001; as cited in Petrides and Furnham, 2006). Future studies should examine the mediating role of such gendered responsibilities in the relationship between trait EI and PSE pursuit in young adults. Additionally, interventions aimed at enhancing trait EI should consider the different types of stressors experienced by men and women.

### Methodological Considerations

A notable methodological strength of the present study was the use of three biennial waves of longitudinal data from a large nationally representative sample of young adults, afforded by the NLSCY database. These design features were not only unique relative to all previous studies of trait EI and PSE outcomes, but also essential in allowing us to investigate the link between trait EI and PSE pursuit in the first place. That said, the correlational nature of the NLSCY data does not permit making causal inferences based on the observed relationships. From the directionality standpoint, higher trait EI may predict future pursuit of PSE, but the PSE experience itself has also been found to predict subsequent gains in trait EI above and beyond the effect of maturation with age (Parker et al., 2005b). In the present study, the mean levels of trait EI (for both men and women) showed a moderate increase from age 20–21 to 24–25. However, this change in trait EI was not significantly moderated by participants' PSE status, suggesting that the observed increase in trait EI was likely due to maturation with age rather than the reciprocal effect of PSE. Still, we cannot rule out the effects of potential third variables unaccounted for in this study.

It should also be noted that the descriptive statistics reported in this study are a decade old (2004–2008), which may not reflect current population statistics and so should be interpreted with reference to its appropriate time period. Nevertheless, the high rates of PSE participation observed in the current sample (e.g., over 60% at age 20–21, increasing to over 70% by age 24–25) is consistent with more recent reports, where Canada has the highest proportion of post-secondary graduates (53%) among all OECD countries (OECD, 2016).

As in all multi-year longitudinal studies, participant attrition and non-response pose a potential limitation to the generalizability of our results. Our missing data analyses showed that participants who had missing cycle-to-cycle data had significantly lower levels of education compared to participants who had complete data for all analyses. This is not surprising, given that indices of lower socioeconomic status are among the most commonly reported predictors of longitudinal survey non-response (Keefer et al., 2013). To account for this potential source of bias, our analyses of change in PSE explicitly controlled for the previous-cycle PSE status. Importantly, no other demographic or trait EI variables were associated with longitudinal or cross-sectional missingness in the current sample. Furthermore, all our analyses were weighted by the NLSCY longitudinal survey weights designed to preserve the original population demographics. These measures would mitigate the biasing impact of longitudinal attrition on our findings. If any, however, the bias would favor null results, resulting in attenuated associations between trait EI and PSE pursuit.

Lastly, the trait EI measure used in the NLSCY (EQ-i: Mini) is a highly abridged version of the EQ-i:S assessment used in previous PSE research (Parker et al., 2011). Although truncating the EQ-i: Mini had permitted the inclusion of trait EI variables in the large NLSCY database, this also likely limited its domain coverage, so its full equivalence to the EQ-i:S cannot be assumed. Indeed, the EQ-i: Mini showed some divergence from the EQ-i:S in the pattern of gender differences across its subscales. At both ages 20–21 and 24–25, women scored significantly higher than men on the Interpersonal subscale of the EQ-i: Mini, which is consistent with gender differences observed for the EQ-i:S (Parker et al., 2011). However, the EQ-i: Mini showed no gender differences on the Adaptability subscale, on which men typically score higher than women based on the EQ-i:S (Parker et al., 2011). It will be important to accrue independent validity evidence for the EQ-i: Mini in future studies, to fully capitalize on these and other findings generated from the NLSCY database.

## Conclusions and Implications

Previous studies on the role of trait EI in PSE attainment have found that individuals who enter PSE with higher levels of trait EI are more likely to earn higher grades, persevere with their studies, and successfully complete their program (Parker et al., 2018). The present study expands on this picture, showing that the role of trait EI in PSE outcomes is evident even earlier in the educational pathway, predicting whether individuals will enter PSE at all during the period of emerging adulthood. This association was especially robust for men, whose PSE participation rates were significantly lower than those of women. The finding that trait EI continued to predict further gains in PSE participation 2 and 4 years later is particularly promising, as it represents an important validation step toward further investment in trait EI interventions, starting with school children. Indeed, integrating socioemotional learning systematically and early in the education cycle, starting with the K-12 curriculum, has been advocated as the best approach for building human capital (Durlak et al., 2011). The economic returns on investing in young people's socioemotional learning throughout K-12 schooling are estimated to be more than 10-fold (Belfield et al., 2015). We would further argue that socioemotional programming should also be part and parcel of student services offered by PSE institutions (Parker et al., 2018). Evidence from controlled intervention studies with school children, undergraduates, and adults supports the efficacy of such efforts in boosting socioemotional traits and improving academic, employment, and other life outcomes (Durlak et al., 2011; Kotsou et al., 2011; Nelis et al., 2011; Dacre Pool and Qualter, 2012; Schutte et al., 2013).

## Author Contributions

All writers were part of the grant for the project (Ontario Human Capital Research and Innovation Fund). HD: main writer, data analysis, research topic. KK: editing, writing, analysis planning. SS: data analysis. RH: editing and research idea. JP: editing and research idea.

### Conflict of Interest Statement

The authors declare that the research was conducted in the absence of any commercial or financial relationships that could be construed as a potential conflict of interest.

## Footnotes

^1^Due to privacy restrictions from Statistics Canada on the release of weighted and unweighted sample sizes, the weighted subgroup sample sizes are not reported in the results. The unweighted total sample size is approximately *N* = 1,400.

## References

[B1] Abada T. Tenkorang E. Y. (2009). Pursuit of university education among the children of immigrants in Canada: the roles of parental human capital and social capital. J. Youth Stud. 12, 185–207. 10.1080/13676260802558870

[B2] Andrei F. Siegling A. B. Aloe A. M. Baldaro B. Petrides K. V. (2016). The incremental validity of the Trait Emotional Intelligence Questionnaire (TEIQue): a systematic review and meta-analysis. J. Pers. Assess. 98, 261–276. 10.1080/00223891.2015.1084630 26457443

[B3] Arnett J. J. (2000). Emerging adulthood: a theory of development from the late teens through the twenties. Am. Psychol. 55, 469–480. 10.1037/0003-066X.55.5.469 10842426

[B4] Austin E. J. Parker J. D. A. Petrides K. V. Saklofske D. H. (2008). Emotional intelligence, in The SAGE Handbook of Personality Theory and Assessment, Vol. 1, eds Boyle G. J. Matthews G. Saklofske D. H. (London: SAGE Publications), 576–596. 10.4135/9781849200462.n28

[B5] Austin E. J. Saklofske D. H. Mastoras S. M. (2010). Emotional intelligence, coping, and exam-related stress in Canadian undergraduate students. Aust. J. Psychol. 62, 42–50. 10.1080/00049530903312899

[B6] Avsec A. (2012). Do emotionally intelligent individuals use more adaptive decision-making styles? Stud. Psychol. 54, 209–220.

[B7] Bakan D. (1966). The Duality of Human Existence. Chicago, IL: Rand McNally.

[B8] Bar-On R. (1997). Bar-On Emotional Quotient Inventory (EQ-i): Technical Manual. Toronto, ON: Multi-Health Systems.

[B9] Bar-On R. (2002). Bar-On Emotional Quotient Inventory: Short (EQ-i:S): Technical Manual. Toronto, ON: Multi-Health Systems.

[B10] Belfield C. Bowden A. B. Klapp A. Levin H. Shand R. Zander S. (2015). The economic value of social and emotional learning. J. Benefit Cost Anal. 6, 508–544. 10.1017/bca.2015.55

[B11] Boerchi D. Magnano P. Lodi E. (2018). Curr. Psychol. 10.1007/s12144-018-9910-y

[B12] Brown C. George C. R. Smith M. L. (2003). The role of Emotional Intelligence in the career commitment and decision-making process. J. Career Assess. 11, 379–392. 10.1177/1069072703255834

[B13] Brown R. F. Schutte N. S. (2006). Direct and indirect relationships between emotional intelligence and subjective fatigue in university students. J. Psychosom. Res. 60, 585–593. 10.1016/j.jpsychores.2006.05.001 16731233

[B14] Carver C. S. Connor-Smith J. K. (2010). Personality and coping. Annu. Rev. Psychol. 61, 679–704. 10.1146/annurev.psych.093008.100352 19572784

[B15] Cotter D. A. Hermsen J. M. Ovadia S. Vanneman R. (2001). The glass ceiling effect. Social Forces, 80, 655–682. 10.1353/sof.2001.0091

[B16] Credé M. Niehorster S. (2012). Adjustment to college as measured by the Student Adaptation to College Questionnaire: a quantitative review of its structure and relationships with correlates and consequences. Educ. Psychol. Rev. 24, 133–165. 10.1007/s10648-011-9184-5

[B17] Dacre Pool L. Qualter P. (2012). Improving emotional intelligence and emotional self-efficacy through a teaching intervention for university students. Learn. Individ. Differ. 22, 306–312. 10.1016/j.lindif.2012.01.010

[B18] Di Fabio A. Blustein D. L. (2010). Emotional intelligence and decisional conflict styles: some empirical evidence among Italian high school students. J. Career Assess. 18, 71–81. 10.1177/1069072709350904

[B19] Di Fabio A. Kenny M. E. (2012). The contribution of emotional intelligence to decisional styles among Italian high school students. J. Career Assess. 20, 404–414. 10.1177/1069072712448893

[B20] Di Fabio A. Palazzeschi L. (2008). Indécision vocationnelle et intelligence émotionnelle: Quelques données empiriques sur un échantillon d'apprentis italiens [Career indecision and emotional intelligence: some empirical evidences in an Italian sample of wage-earning apprentices]. Pratiq. Psychol. 14, 213–222. 10.1016/j.prps.2007.11.006

[B21] Di Fabio A. Palazzeschi L. (2009). Emotional intelligence, personality traits, and career decision difficulties. Int. J. Educ. Vocat. Guid. 9, 135–146. 10.1007/s10775-009-9162-3

[B22] Di Fabio A. Palazzeschi L. Bar-On R. (2012). The role of personality traits, core self-evaluation and emotional intelligence in career decision-making difficulties. J. Employm. Counsel. 49, 118–129. 10.1002/j.2161-1920.2012.00012.x

[B23] Di Fabio A. Saklofske D. H. (2014). Comparing ability and self-report trait emotional intelligence, fluid intelligence, and personality traits in career decision. Pers. Individ. Dif. 64, 174–178. 10.1016/j.paid.2014.02.024

[B24] Durlak J. A. Weissberg R. P. Dymnicki A. B. Taylor R. D. Schellinger K. (2011). The impact of enhancing students' social and emotional learning: a meta-analysis of school-based universal interventions. Child Dev. 82, 474–501. 10.1111/j.1467-8624.2010.01564.x 21291449

[B25] Ferguson C. J. (2009). An effect size primer: a guide for clinicians and researchers. Prof. Psychol. Res. Pract. 40, 532–538. 10.1037/a0015808

[B26] Finnie R. (2012). Access to post-secondary education: the importance of culture. Child. Youth Serv. Rev. 34, 1161–1170. 10.1016/j.childyouth.2012.01.035

[B27] Fussell E. Gauthier A. H. Evans A. (2007). Heterogeneity in the transition to adulthood: the cases of Australia, Canada, and the United States. Eur. J. Popul. 23, 389–414. 10.1007/s10680-007-9136-4

[B28] Gignac G. E. Szodorai E. T. (2016). Effect size guidelines for individual differences researchers. Pers. Individ. Dif. 102, 74–78. 10.1016/j.paid.2016.06.069

[B29] Hill C. J. Bloom H. S. Black A. R. Lipsey M. W. (2008). Empirical benchmarks for interpreting effect sizes in research. Child Development Perspectives, 2, 172–177. 10.1111/j.1750-8606.2008.00061.x

[B30] Humphrey N. Curran A. Morris E. Farrell P. Woods K. (2007). Emotional intelligence and education: a critical review. Educ. Psychol. 27, 235–254. 10.1080/01443410601066735

[B31] Karakuş M. (2013). Emotional Intelligence and negative feelings: a gender specific moderated mediation model. Educ. Stud. 39, 68–82. 10.1080/03055698.2012.671514

[B32] Keefer K. (2015). Self-report assessments of emotional competencies: a critical look at methods and meanings. J. Psychoeduc. Assess. 33, 3–23. 10.1177/0734282914550381

[B33] Keefer K. V. Holden R. R. Parker J. D. (2013). Longitudinal assessment of trait emotional intelligence: measurement invariance and construct continuity from late childhood to adolescence. Psychol. Assess. 25, 1255–1272. 10.1037/a0033903 23914954

[B34] Keefer K. V. Parker J. D. A. Saklofske D. H. (2018). Three decades of emotional intelligence research: Perennial issues, emerging trends, and lessons learned in education—introduction to emotional intelligence in education, in Emotional Intelligence in Education: Integrating Research With Practice, eds Keefer K. V. Parker J. D. A. Saklofske D. H. (New York, NY: Springer), 1–19. 10.1007/978-3-319-90633-1

[B35] Keefer K. V. Parker J. D. A. Wood L. M. (2012). Trait emotional intelligence and university graduation outcomes: using latent profile analysis to identify students at-risk for degree non-completion. J. Psychoeduc. Assess. 30, 402–413. 10.1177/0734282912449446

[B36] Kotsou I. Nelis D. Grégoire J. Mikolajczak M. (2011). Emotional plasticity: Conditions and effects of improving emotional competence in adulthood. J. Appl. Psychol. 96, 827–839. 10.1037/a0023047 21443316

[B37] Krieshok T. Black M. McKay R. (2009). Career decision making: the limits of rationality and the abundance of non-conscious processes. J. Vocat. Behav. 75, 275–290. 10.1016/j.jvb.2009.04.006

[B38] Linnenbrink E. A. (2007). The role of affect in student learning: a multi-dimensional approach to considering the interaction of affect, motivation, and engagement, in Emotion in Education, eds Schutz P. A. Pekrun R. (San Diego, CA: Elsevier Academic Press), 107–124. 10.1016/B978-012372545-5/50008-3

[B39] Lüdtke O. Roberts B. W. Trautwein U. Nagy G. (2011). A random walk down University Avenue: life paths, life events, and personality trait change at the transition to university life. J. Pers. Soc. Psychol. 101, 620–637. 10.1037/a0023743 21744977 PMC3596260

[B40] Mikolajczak M. (2009). Moving beyond the ability-trait debate: a three level model of emotional intelligence. Electron. J. Appl. Psychol. 5, 25–31. 10.7790/ejap.v5i2.175

[B41] Mikolajczak M. Avalosse H. Vancorenland S. Verniest R. Callens M. van Broeck N.. (2015). A nationally representative study of emotional competence and health. Emotion 15:34. 10.1037/emo0000034 25893449

[B42] Mikolajczak M. Van Bellegem S. (2017). Increasing emotional intelligence to decrease healthcare expenditures: how profitable would it be? Pers. Individ. Dif. 116 343–347. 10.1016/j.paid.2017.05.014

[B43] Moulin S. Doray P. Laplante B. Street M. C. (2013). Work intensity and non-completion of university: longitudinal approach and causal inference. J. Educ. Work 26, 333–356. 10.1080/13639080.2011.653554

[B44] Nelis D. Kotsou I. Quoidbach J. Hansenne M. Weytens F. Dupuis P.. (2011). Increasing emotional competence improves psychological and physical well-being, social relationships, and employability. Emotion 11, 354–366. 10.1037/a0021554 21500904

[B45] Nes L. S. Segerstrom S. C. (2006). Dispositional optimism and coping: a meta-analytic review. Pers. Soc. Psychol. Rev. 10, 235–251. 10.1207/s15327957pspr1003_3 16859439

[B46] OECD (2016). Education at a Glance 2016: OECD Indicators. Paris OECD Publishing.

[B47] Parker J. D. A. Duffy J. Wood L. M. Bond B. J. Hogan M. J. (2005a). Academic achievement and emotional intelligence: predicting the successful transition from high school to university. J. First Year Exp. Stud. Transit. 17, 67–78.

[B48] Parker J. D. A. Hogan M. J. Eastabrook J. M. Oke A. Wood L. M. (2006). Emotional intelligence and student retention: predicting the successful transition from high school to university. Pers. Individ. Dif. 41, 1329–1336. 10.1016/j.paid.2006.04.022

[B49] Parker J. D. A. Keefer K. V. Wood L. M. (2011). Toward a brief multidimensional assessment of emotional intelligence: psychometric properties of the Emotional Quotient Inventory—short form. Psychol. Assess. 23, 762–777. 10.1037/a0023289 21500919

[B50] Parker J. D. A. Saklofske D. H. Keefer K. V. (2016). Giftedness and academic success in college and university: why emotional intelligence matters. Gifted Educ. Int. 33, 183–194. 10.1177/0261429416668872

[B51] Parker J. D. A. Saklofske D. H. Wood L. M. Eastabrook J. M. Taylor R. N. (2005b). Stability and change in emotional intelligence: exploring the transition to young adulthood. J. Individ. Differ. 26, 100–106. 10.1027/1614-0001.26.2.100

[B52] Parker J. D. A. Summerfeldt L. J. Hogan M. J. Majeski S. A. (2004). Emotional intelligence and academic success: examining the transition from high school to university. Pers. Individ. Dif. 36, 163–172. 10.1016/S0191-8869(03)00076-X

[B53] Parker J. D. A. Taylor R. N. Keefer K. V. Summerfeldt L. J. (2018). Emotional intelligence and post-secondary education: what have we learned and what have we missed? in Emotional Intelligence in Education: Integrating Research With Practice, eds Keefer K. V. Parker J. D. A. Saklofske D. H. (New York, NY: Springer), 427–452. 10.1007/978-3-319-90633-1_16

[B54] Pascarella E. T. Terenzini P. T. (2005). How College Affects Students: A Third Decade of Research. San Francisco, CA: Jossey-Bass.

[B55] Perera H. N. DiGiacomo M. (2013). The relationship of trait emotional intelligence with academic performance: a meta-analytic review. Learn. Individ. Differ. 28, 20–33. 10.1016/j.lindif.2013.08.002

[B56] Perera H. N. DiGiacomo M. (2015). The role of trait emotional intelligence in academic performance during the university transition: an integrative model of mediation via social support, coping, and adjustment. Pers. Individ. Dif. 83, 208–213. 10.1016/j.paid.2015.04.001

[B57] Petrides K. V. Frederickson N. Furnham A. (2004). The role of trait emotional intelligence in academic performance and deviant behaviour at school. Pers. Individ. Dif. 36, 277–293. 10.1016/S0191-8869(03)00084-9

[B58] Petrides K. V. Furnham A. (2000). On the dimensional structure of emotional intelligence. Pers. Individ. Dif. 29, 313–320. 10.1016/S0191-8869(99)00195-6

[B59] Petrides K. V. Furnham A. (2001). Trait emotional intelligence: psychometric investigation with reference to established trait taxonomies. Eur. J. Pers. 15, 425–448. 10.1002/per.416

[B60] Petrides K. V. Furnham A. (2006). The role of Trait Emotional Intelligence in a gender-specific model of organizational variables. J. Appl. Soc. Psychol. 36, 552–569. 10.1111/j.0021-9029.2006.00019.x

[B61] Petrides K. V. Pérez-González J. C. Furnham A. (2007). On the criterion and incremental validity of trait emotional intelligence. Cogn. Emot. 21, 26–55. 10.1080/02699930601038912

[B62] Petrides K. V. Sanchez-Ruiz M. J. Siegling A. B. Saklofske D. H. Mavroveli S. (2018). Emotional intelligence as personality: Measurement and role of trait emotional intelligence in educational contexts, in Emotional Intelligence in Education: Integrating Research With Practice, eds Keefer K. V. Parker J. D. A. Saklofske D. H. (Cham: Springer), 49–82. 10.1007/978-3-319-90633-1_3

[B63] Qualter P. Whiteley H. Morley A. Dudiak H. (2009). The role of emotional intelligence in the decision to persist with academic studies in HE. Res. Post Compul. Educ. 14, 219–231. 10.1080/13596740903139255

[B64] Richardson M. Abraham C. Bond R. (2012). Psychological correlates of university students' academic performance: A systematic review and meta-analysis. Psychol. Bull. 138, 353–387. 10.1037/a0026838 22352812

[B65] Ross T. Kena G. Rathbun A. KewalRamani A. Zhang J. Kristapovich P. (2012). Higher Education: Gaps in Access and Persistence Study (NCES 2012-046). U.S. Department of Education, National Center for Education Statistics. Washington, DC: Government Printing Office.

[B66] Rowan-Kenyon H. T. Savitz-Romer M. Ott M. W. Swan A. K. Liu P. P. (2017). Finding conceptual coherence: trends and alignment in the scholarship on noncognitive skills and their role in college success and career readiness, in Higher Education: Handbook of Theory and Research, ed Paulsen M. B. (Cham: Springer International Publishing), 141–179. 10.1007/978-3-319-48983-4_4

[B67] Saklofske D. H. Austin E. J. Galloway J. Davidson K. (2007). Individual difference correlates of health-related behaviours: Preliminary evidence for links between emotional intelligence and coping. Personality and Individual Differences, 42, 491–502. 10.1016/j.paid.2006.08.006

[B68] Saklofske D. H. Austin E. J. Mastoras S. M. Beaton L. Osborne S. E. (2012). Relationships of personality, affect, emotional intelligence and coping with student stress and academic success: different patterns of association for stress and success. Learn. Individ. Differ. 22, 251–257. 10.1016/j.lindif.2011.02.010

[B69] Sanchez-Ruiz M. J. Mavroveli S. Poullis J. A. (2013). Trait emotional intelligence and its links to university performance: an examination. Pers. Individ. Dif. 54, 658–662. 10.1016/j.paid.2012.11.013

[B70] Schutte N. S. Malouff J. M. Thorsteinsson E. B. (2013). Increasing emotional intelligence through training: current status and future directions. Int. J. Emot. Educ. 5, 56–72.

[B71] Shaienks D. Gluszynski T. Bayard J. (2008). Postsecondary Education, Participation and Dropping Out: Differences Across University, College and Other Types of Postsecondary Institutions. Statistics Canada, Culture, Tourism and the Centre for Education Statistics (Ottawa, ON).

[B72] Siegling A. B. Saklofske D. H. Vesely A. K. Nordstokke D. W. (2012). Relations of emotional intelligence with gender-linked personality: implications for a refinement of EI constructs. Pers. Individ. Dif. 52, 776–781. 10.1016/j.paid.2012.01.003

[B73] Statistics Canada (2016). Tuition Fees for Degree Programs, Last updated September 7, 2016. Available online at: http://www.statcan.gc.ca/daily-quotidien/160907/dq160907a-eng.htm (accessed June 30, 2017).

[B74] Statistics Canada (2010a). National Longitudinal Survey of Children and Youth, Cycle 8 (Microdata User Guide). Ottawa, ON: Special Surveys Division, Statistics Canada.

[B75] Statistics Canada (2010b). National Longitudinal Survey of Children and Youth, Cycle 8: Survey Instruments. Ottawa, ON: Special Surveys Division, Statistics Canada.

[B76] Statistics Canada (2017). Table 282-0003–Labour Force Survey Estimates (LFS), by Educational Attainment, Sex and Age Group, Unadjusted for Seasonality, Monthly (Persons Unless Otherwise Noted), CANSIM (database). Available online at: http://www5.statcan.gc.ca/cansim/a26?lang=eng&retrLang=eng&id=2820003&&pattern=&stByVal=1&p1=1&p2=31&tabMode=dataTable&csid= (accessed June 13, 2017).

[B77] Summerfeldt L. J. Kloosterman P. H. Antony M. M. Parker J. D. A. (2006). Social anxiety, emotional intelligence, and interpersonal adjustment. J. Psychopathol. Behav. Assess. 28, 57–68. 10.1007/s10862-006-4542-1

[B78] Thompson B. L. Waltz J. Croyle K. Pepper A. C. (2007). Trait meta-mood and affect as predictors of somatic symptoms and life satisfaction. Pers. Individ. Dif. 43, 1786–1795. 10.1016/j.paid.2007.05.017

[B79] Toutkoushian R. K. Paulsen M. B. (2016). Economics of Higher Education: Background, Concepts, and Applications. New York, NY: Springer 10.1007/978-94-017-7506-9

[B80] Uppal S. (2017). Young Men and Women Without a High School Diploma. Insights on Canadian Society. Statistics Canada Catalogue no. 75-006-X. Ottawa, ON: Statistics Canada.

[B81] Wang L. MacCann C. Zhuang X. Liu O. L. Roberts R. D. (2009). Assessing teamwork and collaboration in high school students: a multimethod approach. Can. J. School Psychol. 24, 108–124. 10.1177/0829573509335470

[B82] Wilcox P. Winn S. Fyvie-Gauld M. (2005). ‘It was nothing to do with the university, it was just the people': the role of social support in the first-year experience of higher education. Stud. Higher Educ. 30, 707–722. 10.1080/03075070500340036

[B83] Witkow M. R. Huynh V. Fuligni A. J. (2015). Understanding differences in college persistence: a longitudinal examination of financial circumstances, family obligations, and discrimination in an ethnically diverse sample. Appl. Dev. Sci. 19, 4–18. 10.1080/10888691.2014.946030 25897194 PMC4401427

[B84] Wood L. M. Parker J. D. A. Keefer K. V. (2009). Assessing emotional intelligence using the Emotional Quotient Inventory (EQ-i) and related instruments, in Advances in the Measurement of Emotional Intelligence, eds Stough C. Saklofske D. H. Parker J. D. A. (New York, NY: Springer). 10.1007/978-0-387-88370-0_4

