# Supplementary material for: Predicting the Pursuit of Post-Secondary Education: Role of Trait Emotional Intelligence in a Longitudinal Study
Source: Front Psychol. 2019 May 24;10:1182. doi: 10.3389/fpsyg.2019.01182 (PMC6543007; doi:10.3389/fpsyg.2019.01182)
Supplement: Supplementary file 1 [file Table_1.DOCX]

Appendix A

The EQ-i: Mini Items

Interviewer quote: *“I will read you 20 brief statements. For each one, please choose the answer that best describes you. There are five possible answers. Choose the answer that seems the best, even if you are not sure. This is not a test; there are no 'right' or 'wrong’ answers.”*

Before reading out each item, the interviewer reads out the following statement: *“Tell me how you feel, think, or act most of the time in most situations*.*”* At the end of each item, the interviewer reads the response categories (Likert Scale options): 1 = Very seldom true or not true; 2 = Seldom true; 3 = Sometimes true; 4 = Often true; 5 = Very often true or true.

EQ_Q01 - INTER1: You are sensitive to the feelings of others.

EQ_Q02 - INTRA1: It’s hard for you to describe your feelings (Reversed item).

EQ_Q03 - STRMG1: You’re impatient (Reversed item).

EQ_Q04 – ADAPT1: You try to see things as they really are, without fantasizing or daydreaming.

EQ_Q05 – General Mood: You’re optimistic about most things you do.

EQ_Q06 – INTER2: You’re good at understanding the way other people feel.

EQ_Q07 – INTRA2: Others think that you lack assertiveness (Reversed item).

EQ_Q08 – STRMG2: You have a bad temper (Reversed item).

EQ_Q09 – ADAPT2: When faced with a difficult situation, you like to collect all the information about it that you can.

EQ_Q10 – General Mood: You believe in your ability to handle most upsetting problems.

EQ_Q11 – INTER3: You care what happens to other people.

EQ_Q12 – INTRA3: You’re unable to express your ideas to others (Reversed item).

EQ_Q13 – STRMG3: It is a problem controlling your anger (Reversed item).

EQ_Q14 – ADAPT3: In handling situations that arise, you try to think of as many approaches as you can.

EQ_Q15 – General Mood: You can stay on top of tough situations.

EQ_Q16 – INTER4: You have good relations with others.

EQ_Q17 – INTRA4: It’s hard for you to make decisions on your own (Reversed item).

EQ_Q18 – STRMG4: You have strong impulses that are hard to control (Reversed item).

EQ_ Q19 – ADAPT4: When trying to solve a problem, you look at each possibility and then decide on the best way.

EQ_Q20 – General Mood: You generally expect things will turn out alright, despite setbacks from time to time.

*Note.* General mood items (EQ_Q05, EQ_Q10, EQ_Q15, and EQ_Q20) were not incorporated into any analyses for they are no longer recognized as part of the trait EI construct.

Adapted from: Statistics Canada (2010b, pp. 356-362).
